# A case of primary aldosteronism with resistant hypertension successfully treated by unilateral adrenalectomy after unsuccessful classification of subtype in adrenal venous sampling

**DOI:** 10.1002/ccr3.2317

**Published:** 2019-08-22

**Authors:** Ryo Nakamaru, Koichi Yamamoto, Satoko Nozato, Kazuhiro Hongyo, Motonori Nagasawa, Hideharu Hagiya, Futoshi Nakagami, Hiroshi Akasaka, Hitomi Kurinami, Yoichi Takami, Yasushi Takeya, Ken Sugimoto, Takeshi Ujike, Motohide Uemura, Norio Nonomura, Hiromi Rakugi

**Affiliations:** ^1^ Department of Geriatric and General Medicine Osaka University Graduate School of Medicine Suita Japan; ^2^ Department of Urology Osaka University Graduate School of Medicine Suita Japan

**Keywords:** adrenal venous sampling, adrenalectomy, hypertension, primary aldosteronism

## Abstract

Despite being an established method to identify the unilateral subtype of primary aldosteronism with an indication of adrenalectomy, adrenal venous sampling sometimes fails primarily due to unsuccessful cannulation to adrenal veins. In such cases, the analysis of clinical findings might help to identify the indication of surgery.

## 1 INTRODUCTION

Primary hyperaldosteronism (PA) is one of the major causes of secondary hypertension and accounts for 4%‐19% of patients with hypertension.^1^, ^2^, ^3^ It is caused by the autonomous secretion of aldosterone, which in turn increases the risk of cardiovascular complications, including coronary artery disease, myocardial infarction, arrhythmia, heart failure, stroke, and transient ischemic attack.^4^, ^5^, ^6^, ^7^ To mitigate such complications, early diagnosis and appropriate therapy are therefore important.^8^, ^9^ As indicated in the guidelines for PA, adrenal venous sampling (AVS) is useful to differentiate whether aldosterone hypersecretion is bilateral or unilateral and to evaluate indications for adrenalectomy.^8^, ^10^, ^11^ However, the subtype classification of PA by AVS sometimes results in failure with unsuccessful cannulation mostly to the right adrenal vein due to anatomical location.^12^, ^13^ Here, we report a case of PA with resistant hypertension successfully treated by unilateral adrenalectomy after unsuccessful subtype classification by AVS.

## 2 CASE PRESENTATION

A 53‐year‐old man was referred to our hospital for evaluation and management of hypertension and hypokalemia. He was diagnosed with hypertension at the age of 40 years, and his blood pressure was poorly controlled with a calcium channel blocker (nifedipine at 40 mg/d). He also had a past medical history of vasospastic angina and was a nonsmoker and social drinker. In addition, he had no family history of cardiovascular diseases. On admission, the patient was 168 cm tall and weighed 59 kg. He was afebrile, with a blood pressure of 161/96 mm Hg and a regular pulse of 61 beats per minute. There was no evidence of lung rales, cardiac murmurs, or an abdominal bruit. His neurological examination was unremarkable. Laboratory tests revealed that his serum creatinine level was 1.1 mg/dL, urea nitrogen level was 19 mg/dL, and serum sodium level was 140 mEq/L. He also exhibited hypokalemia with a serum potassium concentration of 2.9 mEq/L; however, he had no subjective symptoms, including muscle weakness. Plasma renin activity (PRA), plasma aldosterone concentration (PAC), and aldosterone‐to‐renin ratio (ARR) were <0.1 ng/mL/h, 292 pg/mL, and >2920, respectively. Other adrenal hormone levels were within normal limits. A chest radiograph and transthoracic echocardiogram showed no significant findings. An electrocardiogram suggested nonspecific ST‐T wave changes with premature atrial contractions.

**Figure 1:**
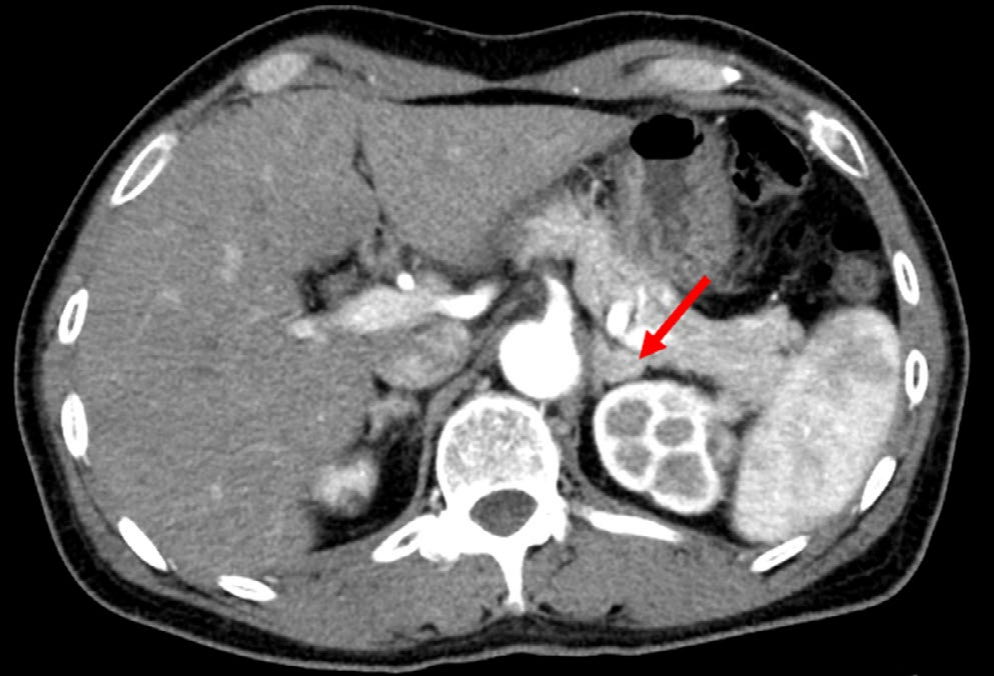
Abdominal computed tomography scan showed an approximate 13 mm left adrenal mass (red arrow)

**Figure 2:**
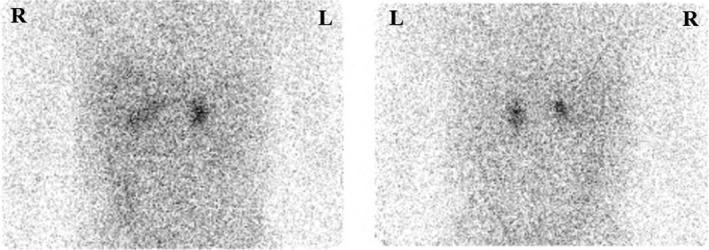
^131^I‐adosterol scintigraphy under dexamethasone suppression showed a bilateral adrenal uptake

Primary hyperaldosteronism was suspected, and therefore, a captopril challenge test and a saline infusion test were performed. All these investigations revealed positive test results (Table 1), which confirmed a diagnosis of PA.^8^ A low‐dose dexamethasone suppression test to exclude the diagnosis of subclinical Cushing's syndrome was negative. An abdominal computed tomography (CT) scan was performed, which showed a left adrenal mass (13 mm) (Figure 1). In adrenocorticotropic hormone (ACTH)‐stimulated AVS, although aldosterone hypersecretion was observed in the left adrenal gland, the lateralization of the disease was not determined due to incomplete cannulation of the right adrenal vein (Table 2). The criteria of successful cannulation were based on the current Expert Consensus Statement.^14^ ^131^I‐adosterol scintigraphy under dexamethasone suppression showed an equivalently increased uptake in both adrenal glands (Figure 2). Because we could not exclude the bilateral PA, including aldosterone‐producing adenoma (APA) with microadenoma in the right adrenal gland, we performed the medical therapy with mineralocorticoid receptor antagonists (MRA). Although we administrated multiple antihypertensive agents including an MRA (eplerenone at 100 mg/d), an angiotensin II receptor blocker (azilsartan at 40 mg/d), a dual action beta blocker/alpha‐1 blocker (carvedilol at 20 mg/d), a calcium channel blocker with an increased dose (nifedipine at 80 mg/d), an alpha‐1 blocker (doxazosin at 2 mg/d), and an alpha‐2 agonist (guanabenz at 4 mg/d), a home reading of the patient's blood pressure was above 140/90 mm Hg. Moreover, he needed a large quantity of potassium chloride to compensate for his hypokalemia.

**Table 1:** Results of confirmatory tests

A. Captopril challenge test			
Times (min)	0	60	90
PRA (ng/mL/h)	0.1	0.1	0.1
PAC (pg/mL)	642.0	351.0	253.0
ARR	6420	3510	2530

B. Saline infusion test		
Times (min)	0	240
PRA (ng/mL/h)	0.1	0.1
PAC (pg/mL)	366.0	286.0
ARR	3660	2860

Abbreviations: ARR, aldosterone‐renin ratio; PA, primary aldosteronism; PAC, plasma aldosterone concentration; PRA, plasma renin activity.

**Table 2:** ACTH‐stimulated adrenal venous sampling

	PAC (pg/mL)	Cortisol (μg/mL)	Selectivity index
The first procedure			
Right adrenal vein	133.0	24.0	0.92
Left adrenal vein	293 000.0	1211.8	46.3
IVC	232.0	26.2	–
The second procedure			
Right adrenal vein	138.0	22.9	0.73
Left adrenal vein	157 000	1104.8	35.4
IVC	1110	31.2	–

Abbreviations: ACTH, adrenocorticotropic hormone; IVC, inferior vena cava; PAC, plasma aldosterone concentration.

**Figure 3:**
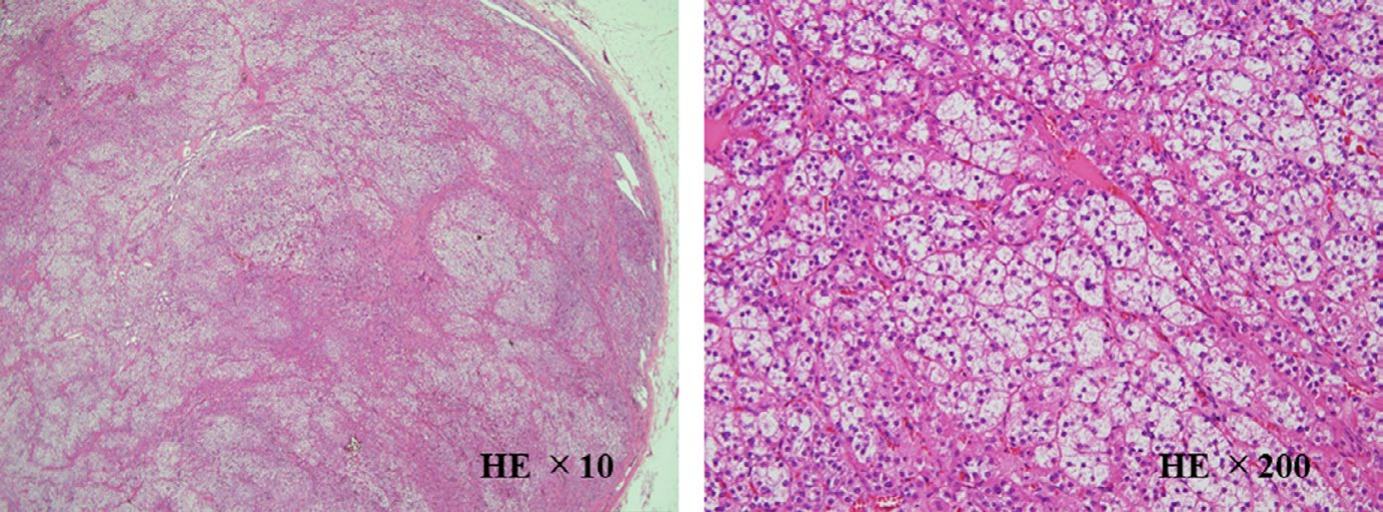
Histopathologic examination of the resected mass showed the presence of tumor cells with clear cytoplasm and small cells with eosinophil cytoplasm by hematoxylin and eosin staining. There was no apparent nuclear atypia, necrosis, and invasion

Despite intensive medical therapy, he underwent percutaneous coronary intervention due to stable angina. Following this, we performed AVS again, but the right adrenal vein was not successfully cannulated (Table 2). We discussed further the indication for surgery and decided to remove the left adrenal gland. After laparoscopic left adrenalectomy, his blood pressure and serum potassium remained normal after the withdrawal of eplerenone, doxazosin, guanabenz, and potassium chloride and a dose reduction of azilsartan (at 20 mg/d), carvedilol (at 10 mg/d), and nifedipine (at 10 mg/d). The PRA and PAC were 0.8 ng/mL/h and 57.1 pg/mL, 3 months after the adrenalectomy, respectively. Macroscopic findings of the resected specimen showed a yellowish colored nodule (13 mm in diameter) in the adrenal gland. We confirmed a diagnosis of an adrenocortical adenoma based on a histopathologic examination (Figure 3); we also confirmed that the tumor cells were rich in the cytoplasm and were eosinophilic without malignant cells.

## 3 DISCUSSION

This is a case of PA with resistant hypertension and severe hypokalemia successfully treated by unilateral adrenalectomy. The subtype of PA was not determined by repeated AVS due to incomplete cannulation to the right adrenal vein. The patient was treated with multiple antihypertensive drugs including MRA, but his blood pressure and hypokalemia could not be controlled. Finally, we decided to perform an adrenalectomy with an expectation of, at least, partial remission of his condition. Consequently, his blood pressure was controlled and complete biochemical success was achieved with the resolution of hypokalemia and normalization of PAC and ARR.^15^

AVS is recommended to differentiate unilateral from bilateral PA and to evaluate indications for surgery.^8^, ^10^, ^11^ Unilateral PA mainly consists of APA, while bilateral PA mainly consists of idiopathic hyperaldosteronism (IHA) and, in some cases, bilateral APA. A recent study reported that the failure rate of AVS is approximately 5%,^15^ the primary reason being the cannulation failure of the right adrenal veins due to its multifarious running direction.^12^ In this case, aldosterone hypersecretion from the left adrenal gland was confirmed by a high PAC/plasma cortisol concentration ratio determined by AVS. Therefore, we identified the following three possible PA classifications for this patient: (1) Left APA, (2) Bilateral APA, and (3) IHA. When we discussed the indication of surgery in this case, we intended to exclude the possibility of IHA, which is poorly improved by adrenalectomy.

Here, we will describe the reasons why the patient did not likely have IHA. First, a left adrenal mass was identified on CT. Minami I et al reported that PA patients with a distinct unilateral adrenal lesion on CT accompanied by hypokalemia, younger age, and poor aldosterone response to renin stimulation could undergo adrenalectomy without AVS.^16^ In addition, PA guidelines in the United States suggest that younger patients (<35 years) with spontaneous hypokalemia, marked aldosterone excess, and unilateral adrenal lesions with radiological features consistent with a cortical adenoma on CT may not need AVS before proceeding to unilateral adrenalectomy.^11^ However, the presence of the left adrenal mass, in this case, was not likely to be a definitive determinant of surgery, because he was relatively older. Second, the patient exhibited severe clinical features of PA with uncontrolled blood pressure and persistent hypokalemia. It is widely recognized that patients with APA have more severe clinical and biochemical findings than those with IHA. It has been shown that most patients with an ARR > 400 have unilateral APA.^16^ Recent studies introduced the prediction score of classifying the PA subtype mainly based on the severity of the disease.^17^, ^18^, ^19^ Using these prediction scores, the possibility of IHA was consistently low in this case. Finally, high PAC after the saline infusion test supported the high probability of APA in the patient. Previous studies reported that PAC after a saline infusion test could predict the difference in classification between APA and IHA.^20^ Given these reasons, we could expect, at least, partial remission of PA by adrenalectomy. As mentioned above, his blood pressure was controlled, and complete biochemical success was achieved after the surgery. Thus, while longer follow‐up will be required to confirm persistent normalization of biochemical parameters, with the overall clinical course, we finally diagnosed a left APA in the patient.

AVS is a gold standard test to confirm the subtype classification of PA. In fact, a recent study reported that patients diagnosed by CT have a decreased likelihood of achieving complete biochemical success compared with a diagnosis by AVS.^21^ Therefore, AVS should basically be performed in most patients with PA who undergo adrenalectomy, if indicated. However, we sometimes experience unsuccessful AVS. In such cases, it is important to analyze accumulated clinical findings to support the classification and lateralization of PA. Such an effort could help in identifying the presence or absence of APA and thus predict the optimal clinical outcome after adrenalectomy.

## CONFLICT OF INTEREST

The authors state that they have no Conflict of Interest.

## AUTHOR CONTRIBUTIONS

RN: designed the study and wrote the initial draft of the manuscript. RN, KY, ST, KH, MN, HH, FN, HA, YT, YT, KS, TU, MU, NN, and HR: contributed to patient management. KY: was a major contributor to the writing of the manuscript. All authors read and approved the final manuscript.

## CONSENT

Written informed consent was obtained from the patient for publication of this case report.
